# Supercritical CO_2_ Antisolvent Fractionation of *Citrus aurantium* Flower Extracts: Enrichment and Characterization of Bioactive Compounds

**DOI:** 10.3390/plants14172678

**Published:** 2025-08-27

**Authors:** Dhekra Trabelsi, José F. Martínez-López, Manef Abderrabba, José S. Urieta, Ana M. Mainar

**Affiliations:** 1 Chemistry Laboratory Materials and Molecules Implementation, Institute for Scientific and Technical Application Studies of La Marsa, Carthage University, Tunis 2070, Tunisia; trabelsi_dhekraa@yahoo.fr (D.T.); mohamedabdelmanef.benabderrabba@ipest.ucar.tn (M.A.); 2 Group of Applied Thermodynamics and Surfaces (GATHERS), IA2 (Agrofood Institute of Aragón), Departamento de Química Física, University of Zaragoza, C/Pedro Cerbuna 12, 50009 Zaragoza, Spain; jfmarti@unizar.es; 3 Group of Applied Thermodynamics and Surfaces (GATHERS), I3A (Aragón Institute of Engineering Research), University of Zaragoza, C/Pedro Cerbuna 12, 50009 Zaragoza, Spain; urieta@unizar.es

**Keywords:** *Citrus aurantium*, supercritical CO_2_, antisolvent fractionation, naringin, neohesperidin, synephrine, quantitative HPLC, ADMET prediction, skin permeability

## Abstract

This study investigates the valorisation of sour orange (*Citrus aurantium* L.) flowers using supercritical antisolvent fractionation (SAF) with CO_2_ as an antisolvent. SAF was applied to selectively recover bioactive compounds from ethanolic extracts, using supercritical CO_2_ to induce precipitation. Response Surface Methodology (RSM) was employed to optimize operational conditions across a pressure range of 8.7–15 MPa and CO_2_ flow rates of 0.6–1.8 kg/h, at a constant temperature of 40 °C. Pressure showed a statistically significant positive effect on precipitate yield, while higher CO_2_ flow rates led to reduced recovery. High-performance liquid chromatography (HPLC) analysis identified naringin (33.7%), neohesperidin (21.6%), and synephrine (9.0%) as the main components of the enriched fractions. SAF enabled the selective concentration of these compounds, supporting its application as a green separation technique. As a complementary evaluation, preliminary in silico predictions of ADMET properties and skin permeability were performed. The results indicated favourable absorption, low predicted toxicity, and limited dermal permeation for the major flavonoids. These findings are consistent with available experimental and regulatory safety data. Overall, the study demonstrates the potential of SAF as an effective green technology for the selective extraction and enrichment of high-value bioactive compounds derived from *Citrus aurantium* flowers, with promising applications in cosmetic, nutraceutical, and pharmaceutical formulations.

## 1. Introduction

Medicinal plants are an important reservoir of bioactive natural products with diverse pharmacological applications. Among them, *Citrus aurantium* L. var. *amara* (sour orange) has drawn increasing interest due to its wide range of therapeutic properties, including antioxidant [1], antitumor [2,3], antimicrobial [4], and radioprotective effects [5]. Extracts from *C. aurantium* have also been used in cosmetics and perfumery—especially its flower-derived essential oil, neroli—and as natural appetite suppressants in weight-loss formulations [6,7].

In recent years, *Citrus*-derived flavonoids and alkaloids have also attracted increasing attention in sustainable agriculture and postharvest management, particularly for their antifungal and preservative properties [8,9,10]. These emerging applications further expand the interdisciplinary value of these compounds.

This broad bioactivity is largely attributed to its rich content of flavonoids (naringin, hesperidin, neohesperidin) and alkaloids (synephrine), which have been extensively studied for their physiological functions. Naringin has demonstrated potent lipid-lowering and antioxidant effects [11,12], while neohesperidin has shown protective action against gastric mucosal lesions [13]. Synephrine, an adrenergic amine structurally related to epinephrine and norepinephrine, is of particular interest in anti-obesity applications [14,15], especially following the FDA ban on ephedrine-containing supplements [16].

More recently, in silico molecular docking studies have proposed neohesperidin and naringin as promising candidates for inhibiting SARS-CoV-2-related targets, including RNA-dependent RNA polymerase and TMPRSS2 [17,18], opening new avenues for repositioning these natural products in antiviral therapy.

In the broader context of natural product recovery, various extraction and purification techniques have been applied, including classical methods such as maceration and Soxhlet [19], as well as modern green techniques like supercritical fluid extraction (SFE) and supercritical antisolvent fractionation (SAF) [20,21]. SFE has been previously used to remove waxes and improve essential oil yields from *C. aurantium* flowers [22]. SAF, on the other hand, uses supercritical CO_2_ as an antisolvent to selectively precipitate target compounds from ethanolic solutions [23]. Although SAF has been successfully applied to other natural matrices [24,25,26], its potential for fractionating *C. aurantium* flower extracts had not been explored prior to this study.

Moreover, recent studies highlight the importance of optimizing SAF parameters—such as pressure, temperature, and flow rates—to tailor the precipitation of compounds with specific biofunctional profiles [27,28]. In this context, integrating computational methods to predict ADMET properties (absorption, distribution, metabolism, excretion, and toxicity) and dermal permeability can greatly assist in evaluating the application potential of these bioactives in pharmaceutical, nutraceutical, and cosmetic formulations [29,30,31]. Recent studies have begun to integrate computational tools with supercritical extraction processes to guide the selection of optimal conditions for obtaining functionally enriched extracts. For example, COSMO-RS and QSAR models have been used to assist in the screening and concentration of antioxidant compounds from *Salvia officinalis* [32], and permeability models have supported extract design for dermocosmetic applications from *Calendula officinalis* [33]. More recently, a combined SFE–in silico approach was applied to ginger herbal dust, using docking and network pharmacology to identify the most promising fractions based on biological targets, and to propose extractive strategies aligned with pharmacological potential [34]. These examples illustrate a growing trend toward hybrid methodologies in extractive research, which this work aims to advance.

The present work explores the use of the SAF technique for the selective precipitation of synephrine, naringin, and neohesperidin from *C. aurantium* flower ethanolic extracts. A response surface methodology (RSM) approach was employed to evaluate the influence of pressure and CO_2_ flow rate on compound recovery. In addition, in silico models were used to assess the pharmacokinetic and dermal absorption profiles of the isolated compounds, providing a holistic insight into their bioavailability and safety.

## 2. Results and Discussion

### 2.1. Precipitate Yield Optimization

In this study, the selection of operating conditions was guided by the vapor–liquid equilibrium (VLE) behaviour of the CO_2_/ethanol system under pressure, ensuring that all experiments remained within the supercritical region. The working temperature was fixed at 40 °C to prevent degradation of thermolabile compounds, in accordance with prior reports [35]. By systematically varying pressure (8.7–15.0 MPa) and CO_2_ flow rate (0.6–1.8 kg/h), the influence of fluid density on the selective precipitation of orange blossom extract components was investigated.

**Figure 1:**
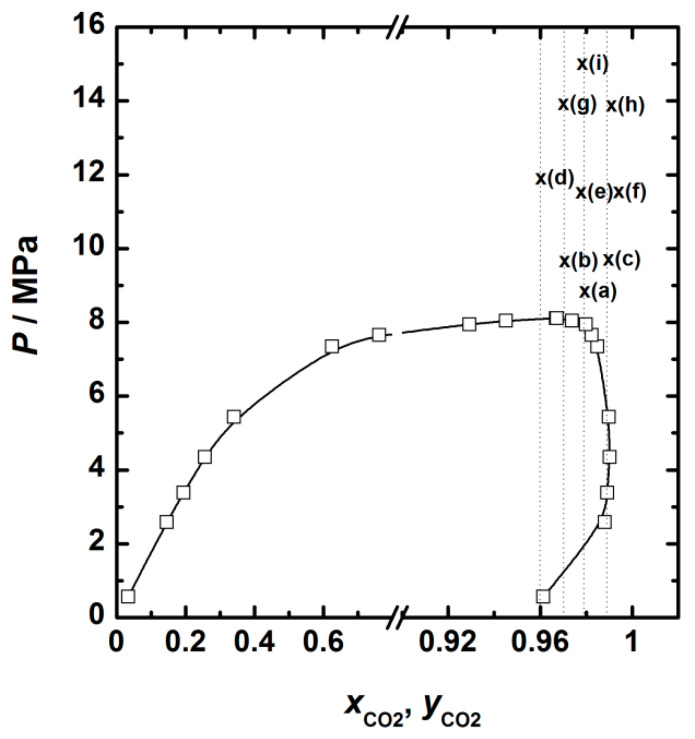
*P*-x,*y* diagram for the binary mixture ethanol-CO_2_ at 40 °C reproduced from Joung et al. [36]. Text indicates the position of the different SAF experiments. (**a**) Experiment 5. (**b**) Experiment 1. (**c**) Experiment 3. (**d**) Experiment 7. (**e**) Experiments 9, 10 and 11. (**f**) Experiment 8. (**g**) Experiment 2. (**h**) Experiment 4. (**i**) Experiment 6.

Figure 1 displays the *P*-*x*_CO_2__-*y*_CO_2__ diagram at 40 °C, based on literature data [36], together with the experimental conditions used in this work. Given the successful results of SAF at approximately 3 wt.% extract concentration in ethanol, the system can be reasonably treated as a pseudo-binary mixture, which simplifies the selection of process conditions based on VLE behavior.

Experimental conditions fell within the single-phase supercritical domain of the CO_2_–ethanol mixture. The observed precipitation yields ranged from 27.4% to 68.2% (Table 1), suggesting strong sensitivity to operating parameters. The experimental results were analyzed using a response surface methodology (RSM) approach. ANOVA confirmed the statistical significance of the model (α < 0.05), with a high coefficient of determination (R^2^ = 0.98) and good predictive capability (adjusted R^2^ = 0.96; predictive R^2^ = 0.87).

The mathematical model (Equation (1)) describes the yield (*Y*) as a function of coded CO_2_ pressure (*x*_1_) and coded flow rate (*x*_2_), including their quadratic effects

*Y* = 53.75 + 5.14 *x*_1_ − 15 *x*_2_ − 3.92 *x*_2_^2^ (1)

It should be noted that *x*_1_ and *x*_2_ represent coded (dimensionless) variables, not the actual pressure and flow rate values. The coding of both factors can be found in Section 3.5, where each level corresponds to a specific real value used in the experimental design. These values were selected according to the Central Composite Design (CCD) approach to allow quadratic modelling of the system behaviour.

Notably, pressure had a significant positive linear effect (*p* < 0.01), while flow rate had a pronounced negative effect (*p* < 0.001). The quadratic effect of flow rate was also significant (*p* < 0.05), as shown in Table 2.

**Figure 2:**
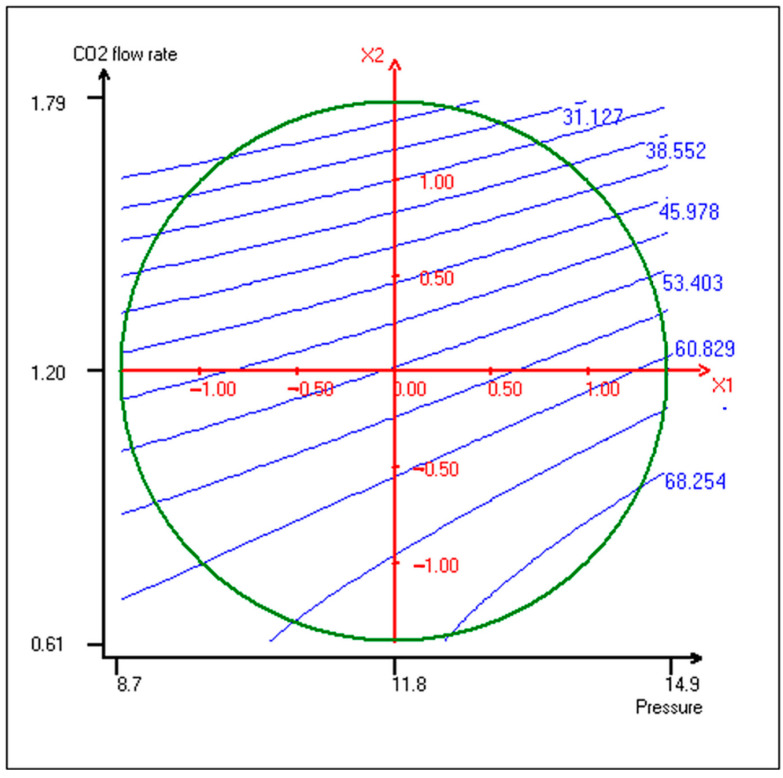
Surface plot of iso-response curves of precipitate yields as function of pressure and CO_2_ flow rate.

To better visualize these effects, Figure 2 presents the iso-response surface plot showing the relationship between CO_2_ pressure, flow rate, and precipitate yield. As illustrated, higher yields were obtained under conditions of high pressure and low flow rate, which aligns with theoretical expectations based on solubility parameters.

The highest yields were obtained at high pressure and low CO_2_ flow rates. This behaviour aligns with the solubility parameter theory: increased pressure near the critical region raises the solubility parameter of CO_2_, enhancing its affinity with ethanol and reducing its solvent power for the target compounds [37,38]. As a result, more solutes are precipitated due to lower ethanol availability for solvation. Conversely, higher flow rates reduce residence time and increase entrainment, which explains the lower precipitation at 1.8 kg/h [39].

The model performed well across the design space, with deviations under 5% in most cases (Table 1). In practical terms, this enables a rational definition of operational “windows of interest” that allow modulation of selectivity according to desired product profiles—whether richer in synephrine, naringin, or neohesperidin—depending on the intended application [40].

### 2.2. Recovery and Quantification of Target Bioactives

The solid collected in the precipitation vessel (PV) was directly analyzed by HPLC, while downstream solutions from the separator (DV) were evaporated prior to analysis. Target compounds were mostly recovered in the filter (PV), with only trace quantities in the DV, confirming their low solubility in the CO_2_–ethanol supercritical mixture under the tested conditions.

As shown in Table 3 the feed solution contained 11.7% synephrine, 25.0% naringin, and 14.9% neohesperidin. After SAF, the combined concentration of these compounds in the precipitate (Table 4) ranged from 33.7% to 62.7%, significantly higher than values reported for other antioxidant-rich matrices. For example, supercritical anti-solvent extraction of grape seed extracts yielded only 1.65–2.37% of target antioxidants [22].

The optimal recovery (62.7%) was obtained at 8.7 MPa and 1.2 kg/h CO_2_ flow rate, with naringin and synephrine reaching 33.7% and 9%, respectively. Interestingly, a CO_2_ flow rate of 1.2 kg/h generally provided favorable conditions for precipitation at various pressures. When increasing flow from 0.6 to 1.2 kg/h at 11.8 MPa (experiments 7 to 9), a modest yield increase was observed. However, further increase to 1.8 kg/h (experiment 8) led to lower recovery, which—despite lying close to the experimental uncertainty—may reflect enhanced solute entrainment by the supercritical fluid [41,42]. When operating at a constant CO_2_ flow rate of 1.2 kg/h (experiments 5, 9, and 6), some variation in total yield was observed as pressure increased from 8.7 to 15.0 MPa. However, given the experimental uncertainty (±4.8%), no clear trend could be conclusively established. The maximum yields of synephrine (6.8 g/kg dry flowers), naringin (22.1 g/kg), and neohesperidin (16.6 g/kg) were obtained under different conditions, reflecting the complex interplay between pressure, flow rate, and solute–solvent interactions. These absolute yields, summarized in Table 5, offer a practical perspective on extract recovery from industrial feedstocks. They enable direct comparison with other extraction processes and are useful for evaluating the scalability of the SAF method.

Compared to conventional methods, the SAF process proved notably effective. Avula et al. [43] reported 1.59 g/kg synephrine from *C. aurantium* extracts via sonication, while the present SAF method achieved over 6 g/kg. Similarly, Giannuzzo et al. [44] reported 14.4 g/kg of naringin from *C. paradisi* peel using supercritical fluid extraction with 15% ethanol, which is surpassed in this work. These results highlight the SAF process as a robust and tunable platform for the selective recovery of bioactive natural products. Its versatility offers a valuable asset for tailoring extracts toward specific biofunctional profiles, particularly relevant for the cosmetic, nutraceutical, or pharmaceutical industries [45,46]. Beyond the context of *Citrus*, the performance of SAF in this work compares favourably with previous applications of this technique to other botanical matrices, such as grape seeds, *Artemisia absinthium*, or *Lavandula luisieri* [22,23,26], where total target compound recoveries were notably lower. Likewise, when considered alongside other green extraction technologies, such as ultrasound-assisted extraction, microwave-assisted extraction, pressurized liquid extraction, or supercritical fluid extraction, SAF offers unique advantages in selectivity and in producing solvent-free precipitates at mild temperatures. These features make it complementary to other methods, particularly in processes where compound stability and downstream solvent removal are critical [10].

### 2.3. SEM Image Analysis and Morphological Characterization

**Figure 3:**
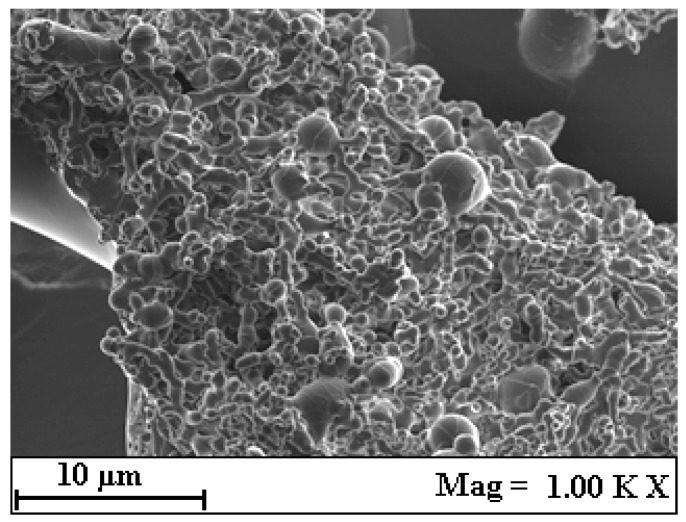
SEM micrograph of the solid collected from the precipitation vessel after SAF. Aggregated, irregular particle morphology is observed, consistent with rapid precipitation under supercritical conditions.

Scanning Electron Microscopy (SEM) was used to analyze the morphology of the solids collected from the precipitation vessel (PV) across different SAF experiments. As a representative case, the results from experiment 5 are shown in Figure 3. In all experiments, the formation of amorphous microparticles was consistently observed. According to Reverchon and De Marco [28], the appearance of amorphous particles can be attributed to the rapid precipitation kinetics typical of supercritical antisolvent (SAS) processes. This rapid nucleation and growth mechanism prevents solute molecules from arranging into a stable crystalline lattice, leading instead to the formation of structurally disordered solid phases. Such behaviour is a hallmark of SAS techniques and is frequently reported in the literature for similar systems.

### 2.4. In Silico ADMET and Skin Permeability Predictions

#### 2.4.1. ADME-Tox Profile of Target Compounds

The predicted ADMET properties for naringin, neohesperidin, and synephrine are summarized in Table 6. The pharmacokinetic and toxicological properties of the three major constituents identified in *Citrus aurantium* extracts—naringin, neohesperidin, and synephrine—were predicted in silico using the admetSAR platform [29]. The estimated LD_50_ values in a rat model ranged between 2.26 and 2.65 mol/kg, placing the compounds in oral toxicity Categories II–III according to the U.S. EPA classification. These values suggest a favourable safety profile, particularly for neohesperidin and naringin, which fall within the less hazardous Category III.

All three compounds were predicted to exhibit good human intestinal absorption (HIA), with synephrine scoring highest. Despite this, none were predicted to cross the blood–brain barrier (BBB), which reduces the likelihood of central nervous system-related side effects—a favourable feature for compounds considered for nutraceutical or topical application.

From a metabolic perspective, neither naringin nor synephrine were identified as substrates or inhibitors of cytochrome P450 enzymes, while neohesperidin showed potential inhibition of CYP3A4. Although such interaction may be of interest in systemic pharmacokinetics, its impact in topical formulations is expected to be negligible due to the compound’s low dermal permeability. Nonetheless, in silico predictions such as these provide a valuable first insight for guiding experimental studies and formulation strategies, particularly when developing multifunctional plant-derived ingredients.

Importantly, none of the three compounds were predicted to be carcinogenic. Moreover, neohesperidin and synephrine tested negative in the Ames test, a standard predictor of mutagenicity. In contrast, naringin yielded a positive Ames prediction, which might warrant further assessment, especially if systemic exposure is expected.

Overall, the ADMET profiles suggest that these bioactives are relatively safe and bioavailable, supporting their use in formulations where oral or topical exposure is expected. In particular, the predicted skin permeability values (log K_p_ < −6.0 cm/s) indicate very low dermal absorption, consistent with effective retention within the stratum corneum—a layer generally considered safe from a toxicological standpoint [30,47].

#### 2.4.2. In Silico Skin Penetration Modelling Results (COSMOperm)

The dermal bioavailability of naringin, neohesperidin, and synephrine was assessed using the COSMOperm module of the COSMOtherm software suite. The applied model represents fully hydrated skin and considers both layered and shunt-based permeation pathways. It should be noted that the COSMOperm predictions assume hydrated and intact skin. Under altered skin conditions, such as dryness or damage, permeability—especially for synephrine—could differ from the values reported here.

Table 7 summarizes the permeability parameters and resistances obtained from Equations (3)–(6), alongside predictive values reported in the literature for comparison.

Among the various compartments, the stratum corneum (SC) exhibited the highest resistance to penetration for all three compounds, reaffirming its role as the primary barrier in transdermal delivery. The calculated log-resistance values for the SC were −8.57 for naringin, −7.96 for neohesperidin, and −5.49 for synephrine. All compounds predominantly followed the transcellular (trans-corneocyte) route, as indicated by significantly higher resistance in the intercellular pathway (*R*_SC,inter_ >> *R*_SC,trans_). The global skin resistance values (*R*_skin_), including both stratified layers and shunt pathways, were estimated as 2.33 × 10^10^ s/m for naringin, 8.80 × 10^9^ s/m for neohesperidin, and 4.20 × 10^8^ s/m for synephrine. The corresponding initial log*K*_p_ permeability coefficients were −8.37, −7.94, and −5.50, respectively. After applying the offset correction proposed in literature (Δlog*K*_p_ = −1.12 cm/s), the final adjusted permeability values were log*K*_p_ = −9.49 (naringin), −9.06 (neohesperidin), and −6.62 (synephrine).

These computational results reinforce the role of the stratum corneum as the dominant and safe barrier [47] in percutaneous absorption and highlight the differing permeability profiles among the evaluated compounds. Notably, synephrine, despite being more permeable overall, remains largely confined to the outer skin layers under simulated conditions, similarly to naringin and neohesperidin. The agreement between COSMOperm predictions and values obtained from alternative in silico models (VEGA [48] and EPI/DERMWIN [49]) supports the reliability of the COSMO-based methodology for early-stage screening of skin-penetrating compounds. This information can be especially valuable in the design of SAF-based processes targeting optimized topical delivery systems for natural bioactives.

Moreover, by linking this permeability and ADMET profiles with the composition of the SAF extracts obtained under different operational conditions, it becomes possible to define “windows of interest” tailored to specific functional applications. For instance, conditions such as 14.1 MPa/0.8 kg·h^−1^ favor synephrine- and naringin-rich compositions, potentially suitable for thermogenic and weight-management formulations [14,15]. Central conditions (11.8 MPa/1.2 kg·h^−1^) promote co-extraction of naringin and neohesperidin, which aligns well with antioxidant and mucosal-protective strategies [13]. Conversely, milder conditions such as 9.6 MPa/1.6 kg·h^−1^ lead to a more selective enrichment in naringin, a compound with reported lipid-lowering and cardiovascular support effects [11,12]. This integrative approach illustrates the potential of SAF not only as a green separation technology but also as a customizable platform to guide product development based on the intended route of administration and target bioactivity.

## 3. Materials and Methods

### 3.1. Chemicals and Reagents

Ethanol (VWR Chemicals, Radnor, PA, USA, 99.96% purity) was used as the solvent for both the maceration process and the preparation of SAF samples. The supercritical antisolvent fractionation (SAF) process employed carbon dioxide (CO_2_) with a purity of 99.8%, supplied by Air Liquide (Paris, France).

Solvents used in the HPLC-PDA analysis included methanol (Scharlab, Barcelona, Spain, 99.9%), Milli-Q water (18.2 MΩ·cm), and glacial acetic acid (VWR Chemicals, Radnor, PA, USA, 99.9%). The analytical standards—naringin, neohesperidin, and synephrine—were all obtained from Sigma-Aldrich (St. Louis, MO, USA).

### 3.2. Feedstock and Sample Preparation

The *Citrus aurantium* flowers used in this study were harvested in Nabeul, a northeastern region of Tunisia. After collection, the flowers were air-dried at room temperature for several weeks until reaching a final moisture content of 8.98% ± 0.11. Moisture was determined using a MA 40 Moisture Analyzer (Sartorius, Göttingen, Germany), with five replicate measurements performed to calculate the standard deviation.

The dried flowers were then ground using an electric grinder, and the resulting powder was sieved to obtain particles with an average diameter of 604 μm. Particle size selection was carried out using a vibratory sieve shaker BA 300N (CISA, Barcelona, Spain), following the ASAE S319.3 standard [50] from the American National Standards Institute. The pretreated material was stored in hermetically sealed, food-grade plastic bags and kept at −20 °C under refrigerated conditions until further use.

### 3.3. Feed Solution Preparation for SAF Experiments

A total of 50 g of ground *Citrus aurantium* flower material was macerated in 0.5 L of absolute ethanol by magnetic stirring for 24 h at room temperature (25 °C). After the extraction period, the mixture was filtered, and the solvent was removed under reduced pressure using a rotary evaporator R-200 (Büchi, Flawil, Switzerland), resulting in a dry crude extract with a mass recovery of 11.7% based on the initial dry plant material (mass_dry extract_/mass _dry plant material_ × 100).

This extract served as the feed solution for the SAF experiments. The feed solution (FS) was prepared by redissolving the dry extract in ethanol to a final concentration of 3% (*w*/*w*). This concentration was selected in line with previous SAS/SAF studies, where moderate values in the range 1–5% (*w*/*w*) have been reported as optimal to balance process stability and productivity [28,51]. In our experience, 3% has consistently provided robust operation without nozzle clogging, while ensuring satisfactory precipitation yields.

### 3.4. Supercritical Antisolvent Fractionation (SAF) Process

SAF experiments were carried out at the Green Chemistry Laboratory (I3A Research Institute, University of Zaragoza) using a laboratory-scale apparatus previously described in the literature [23,26]. The system consisted of a CO_2_ pump (P-SCF), a feed solution (FS) pump (P-LIQ), a 0.5 L precipitation vessel (PV), and a ex low-pressure downstream vessel (DV), all constructed from AISI 316 stainless steel. The pressure within the PV, as well as the temperature and flow rates of both CO_2_ and the feed solution, were automatically controlled.

Liquid CO_2_ was pressurized using a P200 pump (Thar Technologies, Pittsburgh, PA, USA, maximum pressure: 60 MPa), while the FS was delivered through a stainless steel nozzle (100 μm) located at the top of the precipitation vessel using a Waters Series III co-solvent pump (Waters Corporation, Milford, MA, USA, maximum pressure: 40 MPa). The PV was equipped with a bottom filter to collect precipitated solid particles. Supercritical conditions were established in the PV prior to each experiment.

During operation, both supercritical CO_2_ and the liquid feed solution were simultaneously introduced into the PV under tightly controlled flow, temperature, and pressure conditions. The process temperature was maintained at 40 °C to avoid thermal degradation of thermolabile compounds. The feed solution was introduced at a flow rate of 0.45 mL/min, with a concentration of 3% (*w*/*w*), ensuring a CO_2_ molar fraction sufficient to maintain the supercritical state of the (CO_2_ + ethanol) mixture under the selected conditions [52]. The CO_2_ flow rate and PV pressure were systematically varied between 0.6–1.8 kg/h and 8.7–15 MPa, respectively.

Upon contact between the FS and the antisolvent CO_2_, solute precipitation occurred in the PV, resulting in microparticle formation, while soluble components were collected in the downstream vessel (DV). The PV pressure was regulated using an automated backpressure regulator (ABPR, Thar Technologies, Pittsburgh, PA, USA), and the DV pressure was controlled manually via a backpressure regulator (BPR, CIRCOR, Instrumentation Technologies, Houston, TX, USA). All operational parameters—including temperature, pressure, and flow rates—were monitored and controlled using Thar Instruments Process Suite software. The equipment allowed for a maximum pressure of 40 MPa and temperature of 120 °C.

A typical experimental run began by stabilizing the system at the desired operating conditions. Approximately 10 mL of pure ethanol was injected to establish a steady liquid flow before introducing 40 mL of feed solution. Samples from the DV were collected at 5 min intervals. At the end of the process, pure supercritical CO_2_ was flushed through the system for 30 min to eliminate residual solvent. Finally, the system was depressurized, and the microparticles were collected from the PV.

### 3.5. Experimental Design and Statistical Analysis

The supercritical antisolvent fractionation (SAF) process parameters were optimized using Response Surface Methodology (RSM). A Central Composite Design (CCD) was employed to investigate the effects of two independent variables—pressure (*x*_1_) and CO_2_ flow rate (*x*_2_)—on extract yield. The experimental design included three replicates at the central point to ensure reproducibility and estimate experimental error. The selection of variable ranges and levels, as summarized in Table 8, was based on preliminary experimental trials and vapor–liquid equilibrium data for the ethanol–CO_2_ binary system reported in the literature [53,54].

The relationship between the response variable (extract yield, *Y*) and the independent factors was modeled using a second-order polynomial equation (Equation (2)):

*Y* = *b*_0_ + *b*_1_*x*_1_ + *b*_2_*x*_2_ + *b*_11_*x*_1_^2^ + *b*_22_*x*_2_^2^ + *b*_12_*x*_1_*x*_2_ (2)

where *b*_0_ is the intercept, *b*_1_ and *b*_2_ are the linear coefficients, *b*_11_ and *b*_22_ are the quadratic coefficients, and *b*_12_ is the interaction coefficient. The variables *x*_1_ and *x*_2_ represent the coded values of pressure and CO_2_ flow rate, respectively.

The statistical analysis of the experimental results was performed using NemrodW^®^ software (v. 9901, LPRAI, Marseille, France), which enabled model fitting, analysis of variance (ANOVA), and graphical representation of the response surfaces.

### 3.6. Microscopy Observations

The extracts were morphologically characterized using a Field Emission Scanning Electron Microscope (FESEM LEO 1525, Carl Zeiss SMT AG, Oberkochen, Germany). The solid extracts were mounted on carbon tabs previously affixed to aluminum stubs (Agar Scientific, Stansted, UK). Prior to analysis, the samples were sputter-coated with a thin layer of gold–palladium using a sputter coater 108A, (Agar Scientific, Stansted, UK).

### 3.7. Analysis of the Extracts

The concentrations of the compounds present in the extracts were determined by High-Performance Liquid Chromatography (HPLC), using a Waters^®^ Alliance 2695 system coupled with a 2998 Diode Array Detector (Waters Corporation, Milford, MA, USA). UV spectra were recorded over the range of 200–400 nm. Chromatographic separation was carried out on a Waters CORTECS^®^ C18 column (2.7 µm, 4.6 × 150 mm; Waters, Milford, MA, USA) maintained at 30 °C. The mobile phases consisted of (A) water–acetic acid (99.4:0.6, *v*/*v*) and (B) methanol, both HPLC-grade. The elution followed a modified version of the method described by He et al. [55], with the following gradient profile: 0–2 min, 20% B; 2–22 min, 40% B; 22–29 min, 100% B; and 29–39 min, 20% B. The flow rate was 0.6 mL/min, and the injection volume was 15 µL.

**Figure 4:**
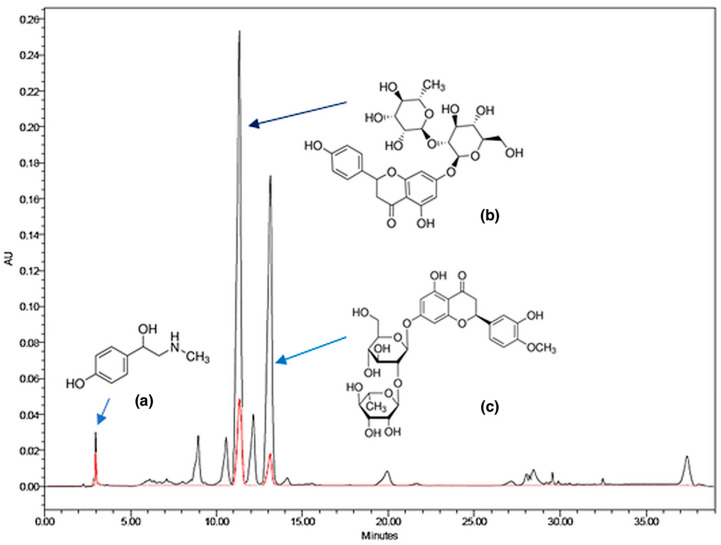
HPLC chromatograms of the SAF precipitate (experiment 6) and reference standards (red). The three target compounds—2.98 min, synephrine (**a**), 11.00 min, neohesperidin (**b**), and 12.8 min, naringin (**c**)—were identified by retention time and UV spectra.

Calibration curves were established using commercial standards of naringin, neohesperidin, and synephrine. Between 2 and 3 mg of each sample—both the feed solution and the collected downstream and filter fractions—were dissolved in 10 mL of pure methanol and filtered through a 0.2 µm nylon Acrodisc^®^ 13 mm syringe filter (Pall Corporation, Port Washington, NY, USA). Detection was performed at 283 nm. Compound identification was based on the comparison of retention times and UV spectra with those of standard compounds and previously reported literature data [55]. The chemical structures of the reference standards are shown in Figure 4.

### 3.8. Computational Methodology: ADMET and Skin Permeation Modelling

#### 3.8.1. In Silico ADME and Toxicity Prediction

To evaluate the pharmacokinetic and toxicological behavior of the selected *Citrus* flavonoids—naringin, neohesperidin, and synephrine—in silico predictions of ADME-Tox properties were performed using the admetSAR online platform [29]. This freely available tool integrates multiple predictive models to estimate key biopharmaceutical properties such as absorption, distribution, metabolism, excretion, and toxicity of bioactive compounds.

#### 3.8.2. Skin Permeation Modelling: COSMOperm Method

To assess the potential application of the antioxidant flavonoids naringin and neohesperidin in the cosmetic industry, their skin permeability was evaluated using the in silico COSMOperm method, as previously described in the literature [30,31]. This model simulates the outermost layer of the skin, the epidermis, considering its differentiation into distinct compartments or layers, each with specific structural characteristics. Such an approach provides a detailed estimation of the dermal permeability of the evaluated compounds, offering insights into their potential topical bioavailability:

- stratum corneum (“SC”; outer horny layer);
- stratum granulosum (“SG”; granular layer, the outermost viable layer);
- stratum spinosum (“SS”; viable prickle layer, releasing neutral barrier lipids);
- stratum basale (“SB”; basal layer, metabolically active;
- appendageal compartment (“shunt”; through the shunts provided by the hair follicles, sweat glands, and sebaceous glands).

The simulated skin consists of a sum of sequential compartment-based resistances for the cell multilayers *R*_stratified cells_, which can be obtained by Equation (3):

*R*_stratified cells_ = *R*_SC_ + *R*_SG_ + *R*_SS_ + *R*_SB_ (3)

Also, diffusion through the shunts of hair follicles and sweat glands takes place. The overall skin model is a parallel resistor model of cellular and shunt pathways obtained as follows:

1/*R*_skin_ = 1/*R*_stratified cells_ + 1/*R*_shunt_ (4)

where *R*_shunt_ is the resistance of the shunt pathway and is kept constant so that, 1/*R*_shunt_ = 2 × 10^−11^ m/s.

Moreover, each cellular compartment i (with i = SC, SG, SS or SB) is considered as a set of transcellular and intercellular pathways, working in parallel, so that the transport of substances can be obtained by means of Equation (5):

1/*R*_i_ = 1/*R*_i,trans_ + 1/*R*_i,inter_ (5)

where *R*_i,trans_ is the mechanism of transcellular absorption (through keratin-corneocytes by partitioning into and out of the cell membrane), and *R*_i,inter_ represents the mechanism of intercellular absorption (trough corneocytes in the lipid-rich extracellular regions).

Finally, the permeability coefficient, *K*_p_, can be calculated, as indicated in Equation (6):

*K*_p_ = 1/*R*_skin_ (6)

where *R*_skin_ is the overall skin resistance obtained by Equation (4)

To carry out all calculations, the 3D chemical structures of the three bioactives were retrieved from the PubChem database. Geometry optimizations were then performed using ab initio quantum chemical methods with Gaussian 09. Density Functional Theory (DFT) was applied, employing the BVP86 functional with the TZVP basis set. The optimized geometries of naringin, neohesperidin, and synephrine used here are provided in the Supplementary Materials.

For the evaluation of skin permeability, the COSMOplex module and the COSMOperm method—both integrated within the COSMOtherm software package—were used. These tools enabled the calculation of the permeability coefficients of each compound, their spatial distribution within the skin model, and the resistance values associated with each compartment.

## 4. Conclusions

The enrichment of bioactive compounds from *C. aurantium* flower by-products was successfully achieved through the Supercritical Antisolvent Fractionation (SAF) process. Naringin, neohesperidin, and synephrine were concentrated up to 33.7%, 21.6%, and 9.0%, respectively, in the solids collected from the precipitation vessel. The optimal experimental conditions for maximum recovery were 15 MPa and 1.2 kg/h for synephrine and naringin, and 8.7 MPa and 1.2 kg/h for neohesperidin. In all cases, the target compounds were efficiently retained in the solid phase, with only trace amounts detected downstream. From 1 kg of dried *C. aurantium* flowers, up to 6.1 g of synephrine, 22.1 g of naringin, and 16.6 g of neohesperidin were recovered, with synephrine levels exceeding those reported using conventional methods.

Complementary in silico studies revealed favorable pharmacokinetic and toxicological profiles for all three compounds. Neohesperidin and synephrine were predicted to be non-mutagenic and non-carcinogenic, and all compounds displayed acceptable oral toxicity profiles. Skin permeability simulations indicated that the compounds exhibit very low dermal absorption, predominantly retained in the stratum corneum, supporting their suitability for topical formulations with minimal systemic exposure.

The COSMOperm skin model predicted very low dermal permeation for all three compounds, with retention primarily in the stratum corneum. This suggests minimal systemic exposure in topical applications and supports their inclusion in cosmetics or dermal products. However, such simulations should be viewed as preliminary estimations, and experimental validation remains essential to confirm their predictive accuracy.

Overall, this study highlights the integrative potential of combining SAF with computational modelling (ADMET, skin permeability) as a rational framework for extract optimization. This approach opens promising avenues for the development of bioactive-rich formulations tailored to cosmetic, nutraceutical, or pharmaceutical applications. These computational predictions offer valuable guidance during early formulation design; however, experimental validation remains necessary to confirm their relevance in complex biological systems. Future work may explore synergistic interactions among compounds, the influence of SAF conditions on their physicochemical properties, and the contribution of formulation systems—such as nanoencapsulation or biopolymeric carriers—to modulate bioavailability, skin permeability, and functional performance.

## Figures and Tables

**Table 1:** Experimental design and results for SAF experiments of *C. aurantium* flowers: Comparison between experimental and predictive values.

Run	Variables		CO_2_ Molar Fraction	*Y*_exp_. (%) ^1^	*Y*_calc_. (%) ^2^	Deviation (%) ^3^
	*x*_1_	*x*_2_				
	CO_2_ Pressure (MPa)	CO_2_ Flowrate (kg/h)				
1	9.6	0.8	0.97	62.0	59.7	1.9
2	14.1	0.8	0.97	68.2	69.8	2.3
3	9.6	1.6	0.99	28.7	29.6	2.9
4	14.1	1.6	0.99	37.3	41.0	9.9
5	8.7	1.2	0.98	46.7	47.4	1.6
6	15.0	1.2	0.98	65.3	62.0	4.9
7	11.8	0.6	0.96	66.9	67.1	0.3
8	11.8	1.8	0.99	27.4	24.7	9.8
9	11.8	1.2	0.98	51.7	53.7	3.8
10	11.8	1.2	0.98	55.3	53.7	2.9
11	11.8	1.2	0.98	54.1	53.7	0.7

^1^ *Y*_exp_. (%), yield of precipitate calculated experimentally. ^2^ *Y*_calc_. (%), yield of precipitate calculated by the model. ^3^$\;Relative\;deviation\;\%=(\frac{Ycalc.}{Yexp.}-1)\times 100$.

**Table 2:** Analysis of coefficients: intercept (*b*_0_), linear effect (*b*_1_, *b*_2_) of cited parameters (pressure and CO_2_ flow rate), respectively, their quadratic effect (*b*_11_, *b*_22_) and their interaction *b*_12_.

Symbol	Coefficient	Inflation Factor	Standard Deviation	t. exp.	Signif. % ^1^
*b*_0_	53.75		1.71	31.44	***
*b*_1_	5.14	1.00	1.04	4.91	**
*b*_2_	−15.00	1.00	1.04	−14.33	***
*b*_11_	0.49	1.09	1.24	0.39	70.8%
*b*_22_	−3.92	1.09	1.24	−3.15	*
*b*_12_	0.59	1.00	1.48	0.40	70.5%

^1^ *** *p* < 0.001; ** *p* < 0.01; * *p* < 0.05.

**Table 3:** Compounds concentration in the feed solution.

Components	Percentage (%) ^1^
Synephrine	11.7
Naringin	25.0
Neohesperidine	14.9

^1^ U_r_ (%) × 100 = ±4.8.

**Table 6:** In silico ADMET-predicted values for the identified natural compounds.

Substance	LD_50_ Rat Model (mol/kg)	HIA	BBB	Carcinogens	Cytochrome p450 Inhibition/Substrate	Oral Acute Toxicity	AMES Toxicity
Naringin	2.2619	HIA^+^ 0.8645	BBB^−^ 0.8414	No	Non-Substrate Non-Inhibitor	Category III	Yes
Neohesperidin	2.4045	HIA^+^ 0.7271	BBB^−^ 0.9396	No	Non-Substrate Only inhibited CYP450-3A4	Category III	No
Synephrine	2.6480	HIA^+^ 0.9943	BBB^−^ 0.9115	No	Non-Substrate Non-Inhibitor	Category II	No

**Table 7:** Detailed permeability parameters for naringin, neohesperidin, and synephrine across epidermal compartments, as predicted by the COSMOperm model. Experimental and calculated log *K*_p_ values from the literature are also included for comparison.

Parameter	Naringin	Neohesperidin	Synephrine
Vehicle	water	water	water
Skin membrane	epidermis	epidermis	epidermis
Rate limiting step	SC via polar transcorneoite pathway	SC via polar transcorneoite pathway	SC via polar transcorneoite pathway
*R*_SC_ (s/m)	3.72 × 10^10^	9.20 × 10^9^	3.07 × 10^7^
*R*_SC,inter_ (s/m)	8.82 × 10^23^	2.99 × 10^20^	4.10 × 10^10^
*R*_SC,trans_ (s/m)	3.72 × 10^10^	9.20 × 10^9^	3.07 × 10^7^
log *K*_p, SC_ (cm/s)	−8.57	−7.96	−5.49
*R*_SG_ (s/m)	1.94 × 10^9^	4.32 × 10^8^	2.92 × 10^5^
log *K*_p, SG_ (cm/s)	−7.29	−6.64	−3.46
*R*_SS_ (s/m)	4.39 × 10^9^	9.80 × 10^8^	6.64 × 10^5^
log *K*_p, SS_ (cm/s)	−7.64	−6.99	−3.82
*R*_SB_ (s/m)	3.04 × 10^8^	6.78 × 10^7^	1.97 × 10^5^
log *K*_p_, _SB_ (cm/s)	−6.48	−5.83	−3.29
*R*_cells_ (s/m)	4.38 × 10^10^	1.07 × 10^10^	3.18 × 10^7^
*R*_shunt_ (s/m)	5.00 × 10^10^	5.00 × 10^10^	5.00 × 10^10^
*R*_skin_ (s/m)	2.33 × 10^10^	8.80 × 10^9^	4.20 × 10^8^
log *K*_p_ (pred.) (cm/s)	−8.37	−7.94	−5.50
log *K*_p_ (pred.) + offset (cm/s)	−9.49	−9.06	−6.62
log *K*_p_ (VEGA) ^1^ (cm/s)	−8.64	−8.66	−6.86
Percent relative deviation ^2^	8.96	4.42	3.62
log *K*_p_ (DERMWIN) ^3^ (cm/s)	−9.91	−9.15	−7.29
Percent relative deviation ^2^	4.43	0.99	10.1

^1^ VEGA HUB—Virtual models for property evaluation of chemicals within a global architecture. ^2^ Percent absolute relative deviation calculated as: $100\left|\frac{{logk}_{p}^{COSMOperm}-{\log k}_{p}^{calc}}{{\log k}_{p}^{COSMOperm}}\right|$. ^3^ Calculated using EPI/DERMWIN 2.0 from U.S. EPA database (in cm/s).

**Table 4:** Experimental conditions and recovery of bioactive compounds in SAF precipitates.

Run	*P* (MPa)	*Q*_CO_2__ (kg/h)	SAF Extracts (%) ^1^			
			Synephrine	Naringin	Neohesperidin	Total ^2^
1	9.6	0.8	5.3	15.9	12.6	33.7
2	14.1	0.8	8.5	27.8	18.2	54.5
3	9.6	1.6	7.2	24.8	11.9	43.9
4	14.1	1.6	8.7	27.5	18.0	54.1
5	8.7	1.2	9.0	33.7	20.0	62.7
6	15.0	1.2	8.0	28.9	21.6	58.5
7	11.8	0.6	6.9	24.4	15.6	46.9
8	11.8	1.8	7.6	26.3	17.9	51.7
9	11.8	1.2	6.4	31.1	18.4	55.9
10	11.8	1.2	8.6	26.4	19.5	54.5
11	11.8	1.2	5,1	27.2	18.1	50.4

^1^ Mass percentage of the target compounds in the precipitate collected on the filter; U_r_ (%) × 100 = ±4.8. ^2^ Sum of the mass percentages of the 3 bioactives collected on the filter.

**Table 5:** Active compound yields (g/kg dry flowers) in SAF experiments.

Run	*P* (MPa)	*Q*_CO_2__ (kg/h)	Compound Yield (g/kg of Dry Flowers) ^1^		
			Synephrine	Naringin	Neohesperidin
1	9.6	0.8	3.8	11.5	9.2
2	14.1	0.8	6.8	22.2	14.5
3	9.6	1.6	2.4	8.3	4.0
4	14.1	1.6	3.8	12.1	7.9
5	8.7	1.2	4.9	18.5	11.0
6	15.0	1.2	6.1	22.1	16.6
7	11.8	0.6	5.4	19.3	12.2
8	11.8	1.8	2.4	8.4	5.7
9	11.8	1.2	4.0	19.4	11.5
10	11.8	1.2	5.6	17.1	12.6
11	11.8	1.2	3.2	17.2	11.5

^1^ U_r_ (%) × 100 = ±4.8.

**Table 8:** Range and levels of independent factors Pressure (*P*) and CO_2_ flow rate (*Q*).

Factors	Symbol	Range and Levels of Independent Factors				
		−1.41	−1	0	1	1.41
(*P*) CO_2_ (MPa)	(*x*_1_)	8.7	9.6	11.8	14.1	15.0
*Q* CO_2_ (kg/h)	(*x*_2_)	0.6	0.8	1.2	1.6	1.8

## Data Availability

The data supporting the reported results are available within the article and its Supplementary Materials.

## Supplementary Materials

The following supporting information can be downloaded at: https://www.mdpi.com/article/10.3390/plants14172678/s1, Optimized geometries of naringin, neohesperidin and synephrine used in permeability calculations.

## Abbreviations

The following abbreviations are used in this manuscript:

**Abbreviation**	**Definition**
ABPR	Automated Backpressure Regulator
ADME	Absorption, Distribution, Metabolism and Excretion
ADMET	Absorption, Distribution, Metabolism, Excretion, and Toxicity
ANOVA	Analysis of Variance
BBB	Blood–Brain Barrier
BPR	Backpressure Regulator
CCD	Central Composite Design
CO_2_	Carbon Dioxide
COSMOperm	Conductor-like Screening Model for Real Solvents—Permeability module
COSMOtherm	Conductor-like Screening Model for Real Solvents—Thermodynamic software suite
DFT	Density Functional Theory
DERMWIN	Dermal Permeability Estimation Program (US EPA)
DV	Downstream Vessel
EPA	Environmental Protection Agency (USA)
FESEM	Field Emission Scanning Electron Microscope
FS	Feed Solution
HIA	Human Intestinal Absorption
HPLC	High-Performance Liquid Chromatography
LD_50_	Lethal Dose for 50% of the population
PV	Precipitation Vessel
QSAR	Quantitative Structure–Activity Relationship
RSM	Response Surface Methodology
SAF	Supercritical Antisolvent Fractionation
SAS	Supercritical Antisolvent
SC	Stratum Corneum
SEM	Scanning Electron Microscopy
SFE	Supercritical Fluid Extraction
TMPRSS2	Transmembrane Protease, Serine 2
TZVP	Triple-Zeta Valence Polarized (basis set in DFT)
UV	Ultraviolet-Visible
VEGA	Virtual models for property evaluation of chemicals
VLE	Vapor–Liquid Equilibrium
