# Supplementary material for: Supercritical CO2 Antisolvent Fractionation of Citrus aurantium Flower Extracts: Enrichment and Characterization of Bioactive Compounds
Source: Plants (Basel). 2025 Aug 27;14(17):2678. doi: 10.3390/plants14172678 (PMC12430218; doi:10.3390/plants14172678)
Supplement: Supplementary file 1 [file plants-14-02678-s001.zip › plants-3806162-supplementary.pdf]

Supporting Information

Supercritical CO_2 Antisolvent Fractionation of *Citrus aurantium* Flower Extracts: Enrichment and Characterization of Bioactive Compounds

Dhekra Trabelsi [1], José F. Martínez-López [2], Manef Abderrabba [1], José S. Urieta [3] and Ana M. Mainar [3,*]

[1] Chemistry Laboratory Materials and Molecules Implementation, Institute for Scientific and Technical
Application Studies of La Marsa, Carthage University, Tunis 2070, Tunisia; trabelsi_dhekraa@yahoo.fr (D.T.); mohamedabdelmanef.benabderrabba@ipest.ucar.tn (M.A.)
[2] Group of Applied Thermodynamics and Surfaces (GATHERS), IA2 (Agrofood Institute of Aragón), Departamento de Química Física, University of Zaragoza, C/Pedro Cerbuna 12, 50009 Zaragoza, Spain; jfmarti@unizar.es
[3] Group of Applied Thermodynamics and Surfaces (GATHERS), I3A (Aragón Institute of Engineering Research), University of Zaragoza, C/Pedro Cerbuna 12, 50009 Zaragoza, Spain; urieta@unizar.es
* Correspondence: ammainar@unizar.es; Tel.: +34-976-761-298

Optimized Geometries of Compounds Used in Permeability Calculations

The following Cartesian coordinates correspond to the optimized geometries (BP-TZVP level) used in the COSMOperm simulations for skin permeability prediction.

Naringin

O	-0.32010000	-1.14520000	-2.50200000
O	0.86200000	-4.19890000	-0.92410000
O	-0.82660000	0.56650000	-3.96840000
O	0.23630000	-2.36370000	0.30250000
O	2.75340000	-3.06530000	-2.89710000
O	-0.71410000	-3.88130000	-3.87750000
O	-0.92930000	1.29240000	-1.18600000
O	2.11050000	2.45100000	-2.98670000
O	0.25030000	4.12750000	-4.28000000
O	1.49050000	-6.93810000	-0.91270000
O	-0.22470000	1.15250000	3.45550000
O	-3.99040000	-0.05200000	0.71310000
O	-4.01260000	2.03650000	2.37340000
O	1.51760000	3.77730000	8.92160000
C	0.73400000	-2.01260000	-2.03100000
C	1.36670000	-2.82840000	-3.20010000
C	0.61710000	-4.17350000	-3.41520000
C	0.16130000	0.11260000	-3.02020000
C	0.12700000	-2.96270000	-0.95660000
C	0.32280000	1.15560000	-1.87880000
C	0.57450000	-5.00330000	-2.09050000
C	0.76800000	2.52050000	-2.47710000
C	-0.21670000	2.94810000	-3.60330000
C	-0.47840000	1.80370000	-4.62920000
C	1.59230000	-6.17420000	-2.12290000
C	0.65310000	1.60450000	-5.67510000
C	-0.59530000	-1.44030000	0.85830000
C	-0.03670000	-0.60380000	1.93070000
C	-1.87130000	-1.24310000	0.47130000
C	-0.78050000	0.35840000	2.50300000
C	-2.18090000	0.56350000	2.08690000
C	-0.81880000	2.33150000	3.92510000
C	-2.70340000	-0.20740000	1.11100000
C	-2.35860000	2.18180000	4.06610000
C	-2.94200000	1.60340000	2.80070000
C	-0.20040000	2.71520000	5.25360000
C	0.14870000	3.99260000	5.50650000
C	0.03230000	1.68990000	6.29000000

C	0.75110000	4.35970000	6.79610000
C	0.58360000	2.02600000	7.46960000
C	0.96150000	3.42280000	7.73840000
H	1.52300000	-1.42360000	-1.55550000
H	1.31750000	-2.24390000	-4.12230000
H	1.12650000	-4.73950000	-4.19990000
H	1.11500000	-0.03470000	-3.52910000
H	-0.91870000	-3.17880000	-1.18140000
H	1.07830000	0.80870000	-1.17010000
H	-0.42290000	-5.43820000	-1.97520000
H	0.74510000	3.27940000	-1.68930000
H	-1.17750000	3.18970000	-3.13570000
H	-1.36670000	2.09570000	-5.20150000
H	2.60650000	-5.78110000	-2.21840000
H	1.37700000	-6.82320000	-2.97470000
H	0.38960000	0.77840000	-6.33820000
H	0.76700000	2.50880000	-6.27490000
H	1.60360000	1.37910000	-5.19460000
H	3.14700000	-3.47320000	-3.69790000
H	-1.11640000	-4.74140000	-4.12500000
H	-0.76110000	1.88940000	-0.42570000
H	2.69190000	2.27890000	-2.21550000
H	0.27910000	4.84080000	-3.60690000
H	2.15350000	-7.65670000	-0.99470000
H	0.96270000	-0.73980000	2.22800000
H	-2.29350000	-1.82660000	-0.29240000
H	-0.61240000	3.11650000	3.19110000
H	-2.80270000	3.15890000	4.26690000
H	-2.59390000	1.50980000	4.89430000
H	-0.00820000	4.74030000	4.78410000
H	-0.23590000	0.68920000	6.11020000
H	-4.36220000	-0.58490000	0.03310000
H	1.01710000	5.36050000	6.97800000
H	0.74350000	1.28790000	8.20080000
H	1.67540000	3.14220000	9.59740000

Neohesperidin

O	-1.46620000	-1.35050000	-2.51650000
O	-1.02000000	-4.33740000	-0.37060000
O	0.73090000	-0.53480000	-2.31670000
O	-0.44500000	-2.17850000	0.06580000
O	-1.66270000	-3.48940000	-4.43360000
O	-2.67310000	-6.03540000	-3.22430000
O	-2.13220000	0.71040000	-4.31290000
O	0.44910000	1.28210000	-5.43930000
O	1.13410000	2.42310000	-2.02040000
O	-1.75590000	-6.75080000	0.87550000
O	1.78010000	1.73390000	1.57020000
O	-2.88640000	1.97980000	0.41610000
O	-1.52700000	4.02810000	1.48440000
O	3.41700000	4.90800000	6.31030000
O	1.64330000	3.48240000	7.70070000
C	-1.09560000	-2.70990000	-2.20830000
C	-1.98300000	-3.67260000	-3.04410000
C	-1.74050000	-5.14240000	-2.59090000
C	-0.42300000	-0.60900000	-3.17960000
C	-1.32160000	-2.96570000	-0.69070000
C	-0.97720000	0.81250000	-3.46400000
C	-1.90890000	-5.25130000	-1.04610000
C	0.12420000	1.70460000	-4.10390000
C	1.40360000	1.67670000	-3.22140000
C	1.83270000	0.22380000	-2.85390000
C	-1.58260000	-6.68410000	-0.54720000
C	2.55620000	-0.52660000	-4.00420000
C	-0.56300000	-0.86120000	0.39230000
C	0.65620000	-0.18010000	0.85290000
C	-1.70500000	-0.15410000	0.28100000
C	0.64320000	1.13640000	1.12330000
C	-0.60160000	1.91150000	0.97340000
C	1.88170000	3.06620000	1.99600000
C	-1.73130000	1.28870000	0.58040000
C	0.83830000	3.99780000	1.31700000
C	-0.52280000	3.35300000	1.25650000
C	1.79920000	3.15660000	3.51050000
C	2.63410000	3.97390000	4.18330000
C	0.81700000	2.36450000	4.27530000
C	2.56980000	4.07940000	5.65120000
C	0.75520000	2.45880000	5.61490000
C	1.67020000	3.35530000	6.34550000

C	0.78490000	2.81630000	8.57500000
H	-0.04550000	-2.90700000	-2.44030000
H	-3.03320000	-3.41230000	-2.88270000
H	-0.72130000	-5.43740000	-2.85670000
H	-0.17620000	-1.10400000	-4.12050000
H	-2.35550000	-2.75000000	-0.41510000
H	-1.28550000	1.25890000	-2.51390000
H	-2.94460000	-5.02510000	-0.77630000
H	-0.23670000	2.73640000	-4.15280000
H	2.21490000	2.17630000	-3.75810000
H	2.56780000	0.30010000	-2.04460000
H	-0.54990000	-6.93570000	-0.79880000
H	-2.25290000	-7.40320000	-1.02260000
H	2.86640000	-1.51210000	-3.65230000
H	3.44330000	0.03210000	-4.30730000
H	1.90640000	-0.65250000	-4.86840000
H	-2.35900000	-3.95050000	-4.94660000
H	-2.41230000	-6.09260000	-4.16760000
H	-2.51060000	1.61380000	-4.37300000
H	-0.34410000	1.45190000	-5.99000000
H	1.98960000	2.50710000	-1.54760000
H	-1.53490000	-7.67280000	1.12790000
H	1.54530000	-0.72970000	0.96870000
H	-2.58770000	-0.61640000	-0.05060000
H	2.87300000	3.41240000	1.68640000
H	1.16180000	4.20500000	0.29440000
H	0.78070000	4.94570000	1.85600000
H	3.34070000	4.55090000	3.65970000
H	0.15290000	1.71960000	3.77930000
H	-3.67470000	1.55690000	0.12580000
H	0.04570000	1.88500000	6.13590000
H	4.05410000	5.42470000	5.85000000
H	1.00810000	3.12940000	9.59570000
H	0.92990000	1.73810000	8.49610000
H	-0.25210000	3.06600000	8.34730000

Synephrine

O	-2.29110000	-0.48120000	-0.20610000
O	4.06770000	-0.57080000	-0.22390000
N	-3.27260000	1.28920000	1.68170000
C	-1.46550000	0.64780000	0.10150000
C	-1.80740000	1.14510000	1.52590000
C	-0.00080000	0.28730000	-0.01730000
C	0.46330000	-1.00300000	0.28590000
C	0.93250000	1.26810000	-0.39020000
C	1.82490000	-1.30810000	0.21850000
C	2.29400000	0.96410000	-0.45590000
C	2.72950000	-0.31880000	-0.15040000
C	-3.62360000	2.63450000	2.12390000
H	-1.71350000	1.42330000	-0.63410000
H	-1.26600000	2.07610000	1.73910000
H	-1.46870000	0.40800000	2.26590000
H	-0.24310000	-1.77960000	0.57190000
H	0.60510000	2.27680000	-0.63410000
H	-3.58310000	0.62850000	2.39750000
H	2.15460000	-2.31470000	0.45470000
H	3.01110000	1.72630000	-0.74560000
H	-3.17880000	-0.19890000	0.09340000
H	-3.16660000	2.87110000	3.09050000
H	-4.70930000	2.70940000	2.23730000
H	-3.31650000	3.38240000	1.38590000
H	4.21730000	-1.50520000	-0.00530000
